# A theoretical epidemiological investigation into the transmission of respiratory infectious diseases during group meals among military personnel based on an individual-based model

**DOI:** 10.3389/fpubh.2025.1545938

**Published:** 2025-05-21

**Authors:** Zuiyuan Guo, Kun Liu, Huawei Jiang, Di Yu, Guangquan Xiao, Yayu Wang, Wei Cui, Jiangfan Li, Jing Tian, Yimin Yang, Feng Wang

**Affiliations:** ^1^The First Department of Infectious Disease Prevention and Control, Center for Disease Control and Prevention in Northern Theater Command, Shenyang, China; ^2^Department of Epidemiology, School of Public Health, Air Force Medical University, Xi’an, China; ^3^Unit 30, Troop 93286 of the Chinese People’s Liberation Army, Shenyang, China; ^4^Department of Medical Protection, Center for Disease Control and Prevention in Northern Theater Command, Shenyang, China; ^5^Department of Orthopedics, General Hospital of Northern Theater Command, Shenyang, China; ^6^Department of Critical Care Medicine, The First Hospital of Jilin University, Changchun, China; ^7^Department of Pest Control and Medical Infection Control, Center for Disease Control and Prevention in Northern Theater Command, Shenyang, China

**Keywords:** respiratory infectious diseases, individual-based model, military, buffet, queues

## Abstract

**Introduction:**

Within military settings, soldiers are arranged to eat together in a self-service manner for every meal. The process of food selection and consumption often leads to close contact amongst individuals, heightening the risk of respiratory infectious disease transmission. To comprehend the transmission dynamics during communal dining, we have conducted an in-depth epidemiological investigation.

**Methods:**

The dining process was divided into two phases: lining up for food and dining at designated seats. Soldiers were randomly split into two queues and entered the food selection area from the same side. The movements of the soldiers dynamically altered both the queues and the contact duration and distance between susceptible individuals and infection sources. We utilized a random computer model using MATLAB software, with the individual as the unit of study, for simulating the food selection process. This model quantitatively analyzed the dynamic process of disease transmission within the queues due to the dispersion of small pathogen-carrying particles.

**Results:**

Our findings indicate that close interactions between individuals during picking up food can result in the persistent transmission of airborne infectious diseases. Implementing measures such as discontinuing buffet-style meals, implementing staggered dining schedules, and mandating mask-wearing during food collection could help control disease transmission during an epidemic.

**Discussion:**

This study demonstrates that the individual-based model can simulate the dynamic process of disease transmission through complex behavioral activities and is more suitable for conducting research on the dynamics of infectious diseases in small-scale populations. Since this is a simulation conducted in a virtual scenario, the results of the model still need to be verified through field investigations. Nevertheless, once robust outbreak investigation studies have yielded reliable model parameters, these parameters can be adapted to this and other similar situations to demonstrate the potential for transmission.

## 1 Introduction

Several experiments have confirmed that respiratory infectious disease viruses, such as those causing influenza, measles, and novel coronavirus, can adhere to droplets and aerosols, spreading externally through actions like coughing and breathing. This consequently leads to the inhalation and subsequent infection of susceptible individuals who are in close contact (1–4). Typically, virus-laden droplets (diameter > 100 μm) can only disperse within a distance of less than 1 meter and settle to the ground within a few seconds, whereas aerosols (diameter < 100 μm) can disperse over a distance of 1–2 meters and remain suspended in the air for a longer period of time (5). If individuals are in constant motion and frequent contact, certain respiratory pathogens may persistently transmit and spread. According to various literature reports, public areas with high human traffic, such as schools, subway stations, airplanes, and hospitals, are accelerators for the rapid spread of respiratory infectious diseases, often leading to outbreak instances (6–10). In military settings, the concentrated living, training, and dining conditions can easily trigger outbreaks of infectious diseases such as influenza, adenovirus, and tuberculosis (10–12). This suggests that continuous close contact among people is a prerequisite for the prevalence of respiratory infectious diseases.

Restaurants serve as congregating points for individuals. According to military management regulations, soldiers are required to have buffet-style meals within predetermined timeframes. Soldiers pick up food in the order in which they arrive at the restaurant. During this process, individuals engage in close contact, increasing the chance of infection via inhalation of droplets or aerosols containing pathogens. Moreover, the dynamic variation in contact can result in expanded contact range, fostering optimal conditions for the transmission of respiratory infectious diseases. Based upon the author’s practical experience, mess halls are critical locales for the transmission of respiratory infectious diseases within military settings. Consequently, establishing a dynamic model of communal dining in the military and quantitatively analyzing the epidemiological distribution patterns bear significant importance in comprehending the disease transmission mechanism within these settings, thereby facilitating targeted epidemic prevention and control. Nonetheless, there is currently a dearth of research reports on this subject matter. Aimed at delving deeper into this issue, we sought to construct a dynamic model of infectious diseases, accurately replicating human behavior during communal meals. We sought to explore the transmission dynamics of specific infectious diseases, analyze the diseases’ epidemiological distribution traits, and provide a theoretical epidemiological foundation for establishing targeted prevention and control measures.

The traditional differential equation model is built on the premise that the population mixes homogeneously and individuals have equal opportunities for contact, thus making it more suitable for macro-level analysis. However, as people continuously change their positions while queuing to collect food, the contact time, duration, and distance between each infectious source and close contacts are in a state of dynamic flux. Consequently, this process cannot be described by differential equations and necessitates the design of a model from a micro-level perspective, with individuals as the unit of study. This involves using computer programming to simulate each person’s behavioral activities and disease progression. Practical instances have demonstrated that, compared to traditional models, this approach can simulate complex social activities more accurately. These activities range from subway network operations, apartment escalator movements, intercity transportation networks, to family gatherings during the Spring Festival (13–16). Within certain model constraints, we first randomly simulated specific behaviors of individuals, from lining up, picking up food, to dining and stored individual, temporal, and spatial information into corresponding data frames. Subsequently, based on this information, identify each infectious source along with all of their close contacts, and gather details regarding the time, duration, and distance of their interactions. Finally, comprehensively utilize this data to calculate the infection probability, determine whether the contacts have been infected, and, if so, ascertain their infection time. Because of the shifting positions in the queues, contact updates occurred continually, leading to consistent emergence of newly infected individuals and disease transmission within the population. As the count of susceptible individuals gradually decreased, the rate of disease transmission would slow and eventually cease.

This study presents an in-depth analysis of the epidemiological characteristics of infectious diseases among military personnel during group food collecting and dining routines. Summarizing, it possesses three key characteristics: (1) Representativeness of the study conclusion—the study’s perspective is grounded in practical reality. While it is applicable specifically to military contexts, it extends as well to other settings like schools, government bodies, and corporations where group buffet dining predominates. (2) Innovation in the study method—due to the dynamic fluctuations in relative positions and contact duration during food selecting, which exhibit both regularity and randomness, it is impracticable to use mathematical equations to characterize individual random behaviors. Consequently, we adopt a random computer modelling method with the individual as the unit of research. (3) Flexibility of the research method—the study ratifies that the flexible application of dynamic models in the form of computer programming and deductive reasoning can more accurately portray the relationship between human activities and disease transmission. It presents a potent research instrument for investigating theoretical epidemiology.

## 2 Materials and methods

### 2.1 Data sources

The study used COVID-19 as a representative of respiratory infectious diseases, and utilized its relative clinical data as parameters for model calculations. All parameters were sourced from existing literature. Additionally, some model parameters were assumptions proposed by the author based on work practice and modelling experience. Specific parameters can be found in Table 1.

**Table 1:** Model parameters.

Description	Distribution characteristics	Numerical values	Sources
Number of classes	Constant	15	Assumed
Number of soldiers per class	Constant	12	Assumed
Number of dishes	Constant	10	Assumed
The distance between consecutive soldiers within the same queue	Constant	0.5 m	Assumed
The distance between two distinct queues	Constant	1 m	Assumed
The distance between two adjacent and opposite soldiers at the same table	Constant	0.8 m	Assumed
Meal times for breakfast, lunch, and dinner on each day	Constant	7:30, 11:30, 17:30	Assumed
Collection duration for each meal item	Uniform distribution	3–8 s	Assumed
Dining duration	Uniform distribution	10–15 min	Assumed
Proportion of susceptible individuals	Constant	0.9	Assumed
Exposure index (*λ*)	Constants	0.01, 0.03, 0.05	Assumed
Incubation period	Lognormal distribution	*μ* = 3.10 days *σ* = 2.60 days	[20]
Infectious duration for mild cases (*d_m_*)	Uniform distribution	6–7 days	[21]
Rate of severe cases (*p_h_*)	Constant	0.29	[22]
Days from onset to isolated treatment (*d_h_*)	Lognormal distribution	*μ* = 3.58 days *σ* = 2.22 days	[22]
Duration of isolated treatment for severe cases	Uniform distribution	9–11 days	[22]

### 2.2 Prerequisites of the model

**Figure 1:**
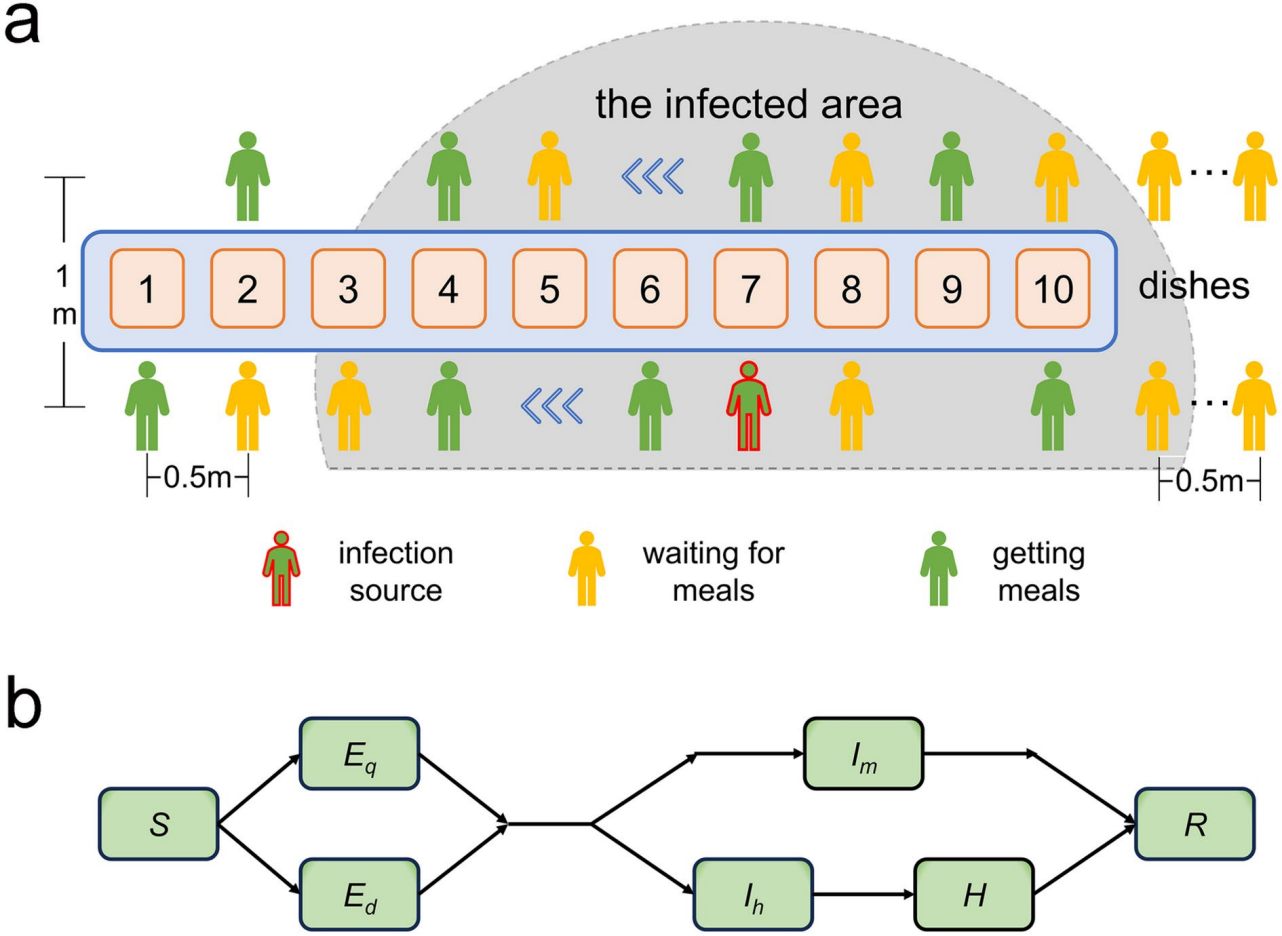
**(a)** Illustration depicting the locations of the two food-collection queues within the pick-up zone and the pathogen-infected area. **(b)** Diagram demonstrating the transition patterns of population infection status. *S* represents susceptible individuals; *E_d_* and *E_q_* denote latent and non-infectious infected individuals that emerge during food collection and dining, respectively; *I_m_* represents cases with mild symptoms during the infectious period; *I_h_* denotes infectious patients with severe symptoms needing time off for treatment or rest; and *R* signifies recoveries.

1. The dining venue was a military mess hall. All soldiers were organized to dine uniformly, excluding patients receiving medical treatment. The daily times for breakfast, lunch, and dinner were set for 7:30, 11:30, and 17:30, respectively. Each meal had 10 food stations (including staple food, side dishes, and fruits). All diners were randomly split into two queues for food serving based on their order of arrival at the food selection area, proceeding in the same direction. Individuals not yet at the food selection area queued to wait their turn. The queue arrangement is depicted in Figure 1a.
2. Each diner served themselves 4 to 8 dishes per meal, and the locations and durations spent on each dish (ranging from 3 to 8 s) were randomly distributed. Diners were not allowed to cross individuals ahead of them during the serving process. Upon serving all their dishes, individuals exited the queue. Each person was allowed only one opportunity to serve themselves food. The distance between two adjacent people in the same queue was 0.5 meters, and the distance between the two queues was 1 meter. The spatial distribution of the food serving area is represented in Figure 1a.
3. The diners were all from a military company, consisting of a total of 15 squads, with each squad comprising of 12 individuals. There was a designated table for every four people for dining with fixed seats. The distance between adjacent or opposite diners was set at 0.8 meters. Each diner’s mealtime was randomly distributed within a 10–15 min window. All diners at the same table departed together after the last individual finished dining. As the distance between different dining tables was greater than 2 meters, disease transmission was prevented.
4. An infectious source can infect susceptible individuals within a distance of 2 meters. Referring to the form of the Wells-Riley equation (Equation 2) (5), we express the contact infection probability, *q*, as a function with the exposure index, *λ*, as a parameter and contact duration, *t*, and contact distance, *d*, as variables:

(1) $$q=1-\exp(-\mathrm{\lambda t}/d^{2})$$

**Figures 2:**
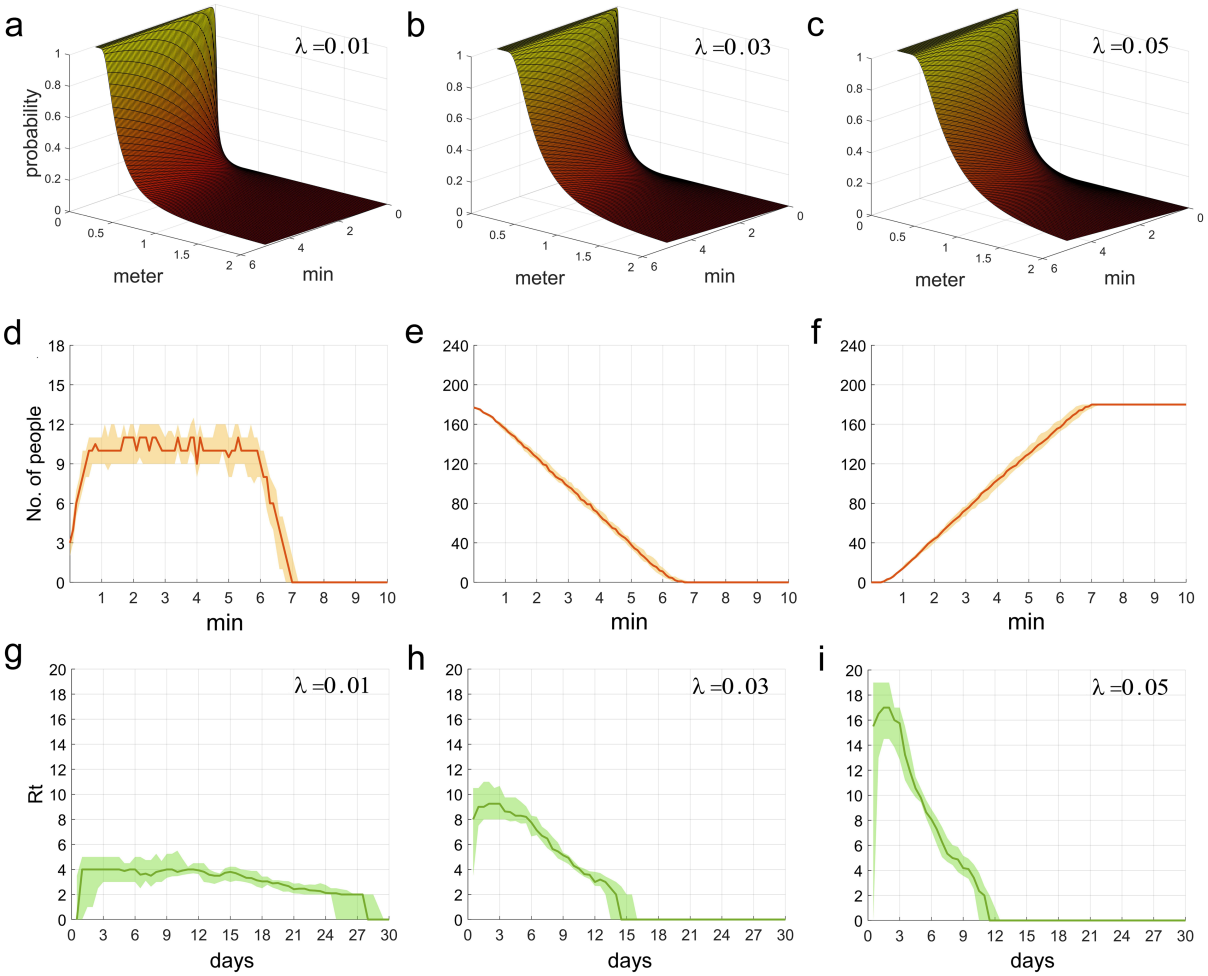
**(a–c)** The functional relationship between the contact infection probability *q*, and the factors of contact duration *t* and distance *d*. **(d–f)** This refers to the time distribution of individuals who are picking up food, waiting for food pickup, and those who have completed food pickup, respectively, in the two queues. **(g–i)** The time distribution of *Rt* during the epidemic period.

We integrated the three parameters from the Wells-Riley equation into a unique parameter, *λ*. *q* is inversely proportional to *d^2^*, indicating that as susceptible individuals progressively approach the infection source, the infection probability increases rapidly. This relationship can be observed in Figures 2a–c. When the distance from the infection source is within 1 meter, the likelihood of infection abruptly augments, which aligns with the spatial distribution properties of droplets (within 1 meter, the primary carriers of respiratory infectious disease pathogens) and aerosols (between 1–2 meters) (5).

1. Susceptible individuals (*S*) who become infected are classified into latent infected individuals, *E_q_* (infected during food collection) and *E_d_* (infected during dining), based on the location of infection. After the incubation period, these individuals progress to become infectious cases. A fraction of these cases (*I_h_*), which exhibit severe symptoms, take leave for treatment or rest and are transferred to a healthcare facility (*H*). During the infectious period, from onset to treatment, *I_h_* maintain normal ingestion behavior and remain infectious. However, they cease to be transmissible while undergoing treatment. Upon recovery, they resume their regular work activities and continue visiting restaurants for meals. Meanwhile, another fraction, *I_m_*, with milder symptoms, manage to maintain their regular work and ingestion patterns throughout their recovery. Recovered individuals (*R*) possess immunity and will not be reinfected. To ensure the specificity of our study, we presume that disease transmission is exclusive to restaurant environments and is inhibited elsewhere. Figure 1b delineates the process entailing variation in the infection statuses of individuals.

### 2.3 Design framework of the model

This study employed a computer program encoded in MATLAB language, which did not incorporate differential equations. Hence, the program’s design framework is introduced purely in a logical sequence.

#### 2.3.1 Basic setup

(1) Set the basic parameters of the model, such as the distance between adjacent individuals in the queue, the proportion of severe cases, the incubation period, and the number of large-scale iterations (see Appendix I, lines 22–46). (2) Initiate 50 repeated large-scale iterations, with each iteration representing the entire process of a disease transmission. The purpose of these iterations is to estimate the median and fluctuation range of the number of infected individuals through multiple repeated calculations. (3) Prior to each major cycle, IDs for all susceptible individuals and one index case are randomly assigned. The index case’s ID, incubation period, infection time, symptom onset time, severity status, and other pertinent details are then stored in the first row of an information data frame (with each row representing a newly infected individual and each column corresponding to the aforementioned details). (4) Subsequently, initiate a loop spanning from day 1 to day 30, with each day further divided into three sub-loops (simulating breakfast, lunch, and dinner, respectively). (5) Before the start of each sub-loop, determine the IDs of all individuals who will be dining at the restaurant. Take into account that individuals in the *I_h_* group are receiving treatment and are therefore unable to dine. Randomly assign meal collection points and meal collection durations for each dining individual, and store this information in a location data frame. The program code for the above procedures is provided in Appendix I, lines 48–126.

#### 2.3.2 Configuration of the food collection queue

(1) In the presence of an infection source and susceptible individuals, diners are randomly divided into two queues. Using the location data frame, the initial food pick-up positions for both queues are selected, documenting information such as the index of each individual picking-up and waiting in the collection zone, their current pick-up location, the time they pick-up at the position, and the succeeding pick-up location. (2) Ascertain the time when the position of the next individual collecting food changes and update the positions of all individuals waiting following the change. The specific procedures entail: a. Identifying the first person in the queue who has finished picking up their meal at a particular location and documenting this time as *t*. b. According to the location data frame and the position distribution of those present in the food collection area, their potential succeeding positions are determined. These positions include moving forward to the next pick-up site, exiting the queue, advancing as a waiting individual, or remaining stationary. If an individual moves forward or exits the queue, it is necessary to determine the position changes of each waiting individual from behind that person to the next person picking up food, and effectuate updates. c. After carrying out the above steps, if there is a vacant spot at position 10, it would be occupied by the next individual in queue. (3) Once the positions are updated, store time *t*, indexes of individuals picking up food and waiting, along with their corresponding positions into a state data frame. (4) Archive the information, including the time post-update at which individuals are picking up food at their respective positions, along with their subsequent food pick-up locations, etc. Terminate the aforementioned operations once all individuals have finished collecting their food. Refer to the corresponding program code in Appendix I, lines 128–388.

#### 2.3.3 Disease transmission during food collection

Based on the state data frame, we are able to calculate the contact duration and average distances between each infection source and all close contacts in the queues, and determine the disease transmission relationship amongst them. (1) When a certain infection source or other individuals in their queue alter their positions, find the indexes, contact duration, and distances of all close contacts with the infection source in the queue according to the state data frame. (2) When the infection source or anyone in the alternate queue changes their position, identify the indexes, contact duration, and distances of all close contacts of the infection source in that other queue. (3) It’s essential to filter this information and consolidate the indexes, cumulative contact duration, and average distances of all susceptible close contacts of the infection source. (4) Utilizing Equation 1, derive the probability of each close-contact individual contracting the infection. Subsequently, generate a random number following the Bernoulli distribution to establish if they have been infected (1 for infected, 0 for not infected). Then, append the information of all infected individuals to the information data frame. Refer to the corresponding program code in Appendix I, lines 390–511.

#### 2.3.4 Disease transmission during dining

(1) Find dining tables where the infection source and susceptible individuals currently both exist, and determine their indexes and seats. (2) Calculate the probability of susceptible individuals infected by each infection source at the same dining table, and generate a random number based on the Bernoulli distribution to determine whether they were infected. (3) Store the infection information of infected individuals into the information data frame. See the above program code in Appendix I lines 554–645.

**Figure 3:**
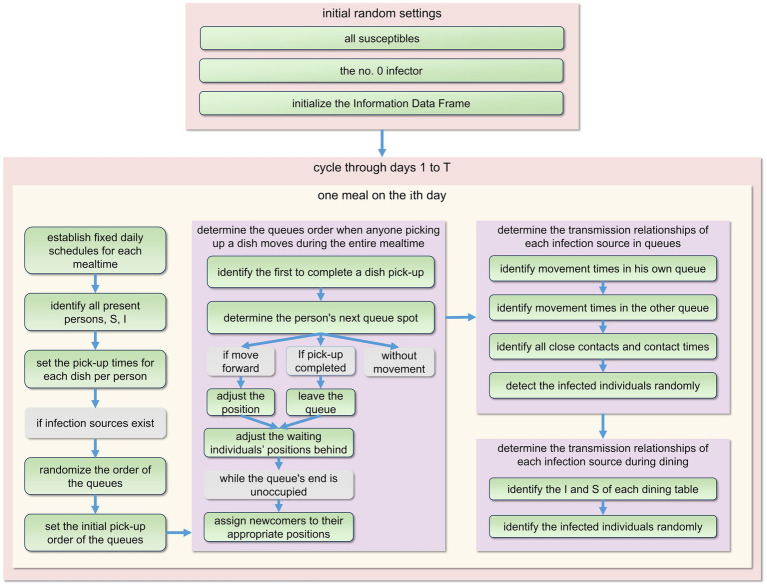
The fundamental structure of the model’s program design during each primary cycle. Every day comprises three meals: breakfast, lunch, and dinner. To prevent repetition, we only present the plan for one meal here. For detailed procedures, please refer to Appendix I.

The program execution continued until day 30. Using the information data frame, the time distribution of susceptible and infected individuals within this simulation was calculated. Upon the completion of 50 primary loops, the median and range of fluctuation of individual count could be derived. The program’s design framework is depicted in Figure 3.

Even though this study did not incorporate any form of differential equation, to more clearly showcase the pattern of population changes amongst those in different infection statuses, a system of equations was employed to convey the quantitative relationship amongst them.


$$\begin{array}{l} {\mathrm{\Delta S}}_{d}=-\sum_{k=1}^{3}\sum_{i=1}^{I}(\sum_{\text{line}1}Q_{1i}^{d}X_{1i}^{d}+\sum_{\text{line}2}Q_{2i}^{d}X_{2i}^{d})-\sum_{k=1}^{3}\sum_{i=1}^{I}\sum_{b}Q_{\mathrm{bi}}^{d}X_{\mathrm{bi}}^{d}\\ {\mathrm{\Delta E}}_{d}=\sum_{k=1}^{3}\sum_{i=1}^{I}(\sum_{\text{line}1}Q_{1i}^{d}X_{1i}^{d}+\sum_{\text{line}2}Q_{2i}^{d}X_{2i}^{d})+\sum_{k=1}^{3}\sum_{i=1}^{I}\sum_{b}Q_{\mathrm{bi}}^{d}X_{\mathrm{bi}}^{d}-{\mathrm{\delta E}}_{d}\\ {\mathrm{\Delta I}}_{d}={\mathrm{\delta E}}_{d}-{\mathrm{\phi p}}_{h}I_{d}-\varphi (1-p_{h})I_{d}\\ {\mathrm{\Delta H}}_{d}={\mathrm{\phi p}}_{h}I_{d}-{\mathrm{\eta H}}_{d}\\ {\mathrm{\Delta R}}_{d}=\varphi (1-p_{h})I_{d}+{\mathrm{\eta H}}_{d} \end{array}$$

Q∼B(1,q) ; X∼ 1 (susceptible) or 0 (not susceptible).

Wherein $△S_{d}$ stands for $S_{d}-S_{d-1}$ , *X_i_* denotes the susceptibility of the *i*-th infectious source’s close contacts, with 1 representing susceptible and 0 representing non-susceptible. *Q_i_* indicates whether a susceptible individual is infected (1) or not (0) after contact with the *i*-th infectious source. $\sum_{\text{line}1}Q_{1\mathrm{ik}}^{d}X_{1\mathrm{ik}}^{d}$ represents the number of close contacts in the same queue as the *i*-th infectious source who were infected by the *k*-th meal (including breakfast, lunch, and dinner) on the *d*-th day caused by the infectious source, $\sum_{b}Q_{\mathrm{bik}}^{d}X_{\mathrm{bik}}^{d}$ represents the number of close contacts at the same table as the *i*-th infectious source who were infected due to the *k*-th meal (on the *d*-th day) served by the infectious source. 1/δ represents the incubation period, 1/ϕ represents the duration of infectiousness for *I_h_*, 1/φ denotes the duration of infectiousness for *I_m_*, *p_h_* denotes the rate of severe cases, and 1/η represents the treatment duration for *I_h_*.

### 2.4 Sensitivity analyses

To narrow down the focus of the study, we retained only the principal components of people’s behavioral activities, resulting in an idealized model. In actuality, the outbreak of infectious diseases forms a complex system, which makes obtaining matching epidemic data for fitting an arduous task. To evaluate the reliability and rationale of the model, we adopted an alternative approach–conducting a sensitivity analysis on six significant parameters within the model: the exposure index (*λ*), the rate of severe cases (*p_h_*), infectious duration of *I_m_* (*d_m_*), infectious duration of *I_h_* (*d_h_*), incubation period, and the proportion of susceptible individuals in the population (*p*).

We incorporated the Partial Rank Correlation Coefficients and Latin Hypercube Sampling (PRCC-LHS) method, a widely-utilized algorithm in sensitivity analyses. This method computes correlations between a parameter set and model outputs upon the exclusion of the linear impacts of the target parameter (17). Each parameter interval was subdivided into *N* smaller and equal intervals, and a sample was arbitrarily selected from each segment. These selected parameter samples were subsequently integrated into the model to compute the outputs at each time point (17, 18). A series of standard coefficients reflecting the correlation between each parameter and the model output were established. For more detailed information, please refer to Appendix II.

All computations were executed using MATLAB R2019a software (MathWorks, Natick, Massachusetts, United States).

## 3 Results

### 3.1 Time distribution of infection probability, queue length, and effective reproduction number

The second row of Figure 2 illustrates the temporal distribution of individuals within the two food collection queues. Figure 2d delineates the fluctuations in the count of individuals picking up food (excluding those waiting). It is observable that the count of individuals collecting food rapidly escalates within the initial minute, then remains at around 10–11, and steeply declines post the 6-min mark until food collection ceases at the 7-min mark. Figure 2e depicts the course of the number of individuals waiting (including those at and behind the food pick-up area). The initial count of individuals waiting was nearly the total of 180, however, as the food collection progressed, people continuously completed their pick-ups and exited the queue, which led to a rapid decline in the count of waiting individuals. Figure 2f displays the trend of the cumulative count of individuals who have completed food collection. It is observably intuitive that the temporal distribution of waiting individuals and those who have finished food collection forms an inclined straight line, with minimal fluctuations. The effective reproduction number, *Rt,* represents the number of secondary infections caused by an infector who becomes infectious at time *t* throughout their entire infectious period. This metric can reflect the current transmission rate of the epidemic. Figures 2g–i illustrate the temporal variation of *Rt* over the period of 0–30 days. It can be observed that the smaller the value of *λ*, the smaller the initial value of *Rt,* leading to a more stable disease transmission rate and a longer epidemic period; conversely, a larger initial value of *Rt* corresponds to a faster decline in the disease transmission rate and a shorter epidemic period. From these figures, it is clear that a larger value of *λ* is associated with a higher probability of infection and a faster speed of epidemic transmission.

### 3.2 Temporal distribution characteristics of the population when the exposure index varies

**Figure 4:**
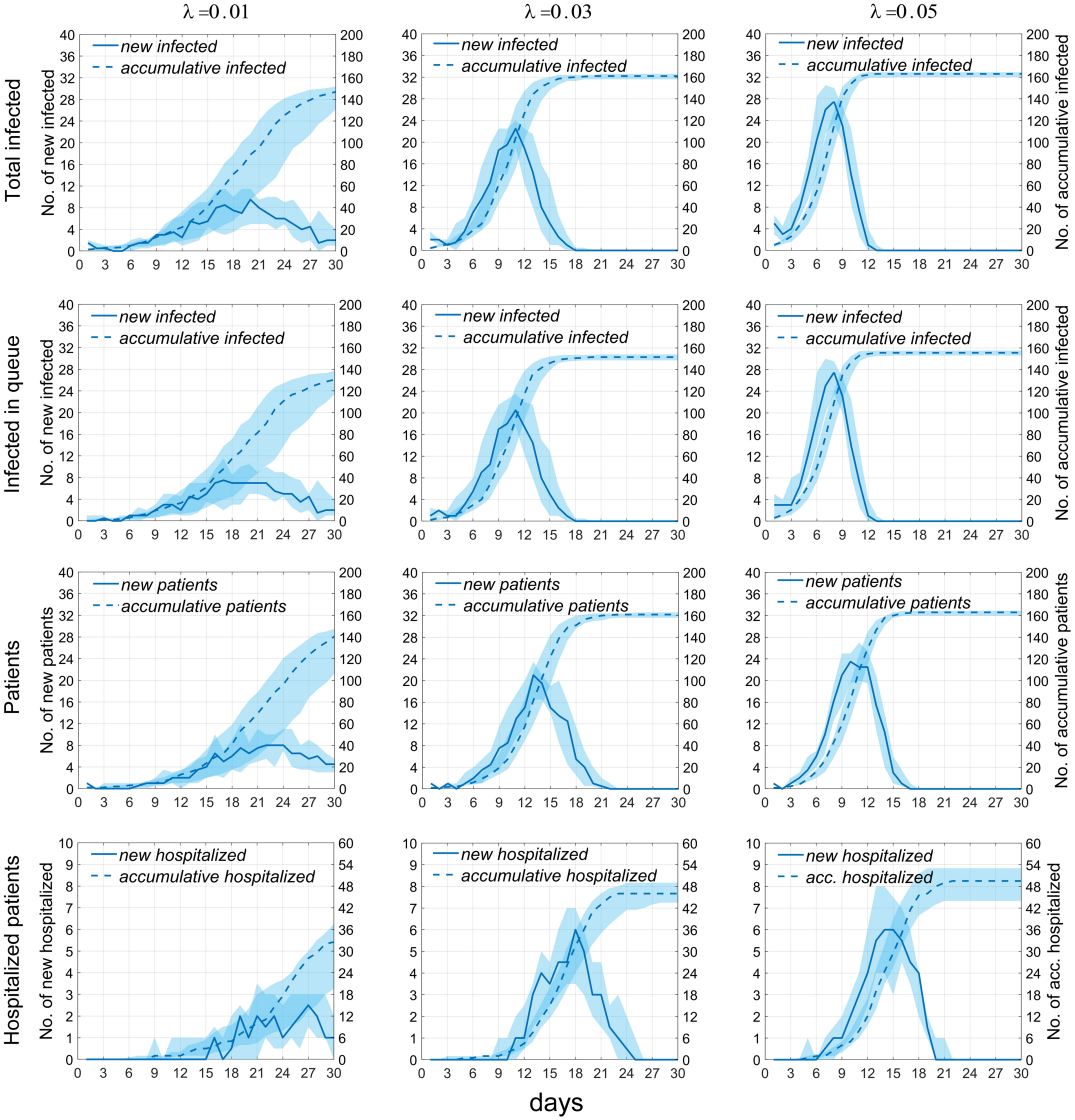
Temporal distribution of the number of infected individuals and cases when the exposure index varies. Rows 1–4, respectively, display the temporal distributions of the number of infections occurring during food pickup and dining, the number of infections occurring specifically during food pickup, the number of cases, and the number of hospitalized patients. The solid blue line represents the median number of new infections or cases, measured by the left vertical axis; the dashed line represents the median cumulative number of infections or cases, measured by the right vertical axis; the blue shaded area indicates the 25–75% fluctuation interval after 50 simulations.

We plotted the time distribution of newly infected individuals, new infectors, and newly treated patients. The first row of Figure 4 shows the time distribution of new and cumulative infected individuals generated during picking up food and dining. To more clearly reflect the impact of queuing for food on disease transmission, the second row of Figure 4 shows the time distribution of infected individuals generated during queuing for food. It can be seen that on day 30, the proportion of infected individuals while queuing for food to the whole infected individuals increased from 79.6% when *λ* = 0.01 to 93.9% when λ = 0.05, indicating that queuing for food is the main factor in disease transmission, moreover, an increase in *λ* will lead to an increase in the total number of infected individuals. The third row of the figure shows the time distribution of infectors (including *I_m_* and *I_h_*). The fourth row shows the time distribution of newly treated patients. It can be seen from the figure that as the contact index *λ* increases gradually, the growth rate of these four groups of people also increases accordingly. The time when the number of newly increased people reaches the peak advances, and the number of people at the time of the peak also increases accordingly. The specific values are shown in Table 2.

**Table 2:** Temporal distribution of new and accumulative infected individuals, infectors, and patients under treatment.

Epidemic-relatedStatistics	λ = 0.01		λ = 0.03		λ = 0.05	
	Peak number of people	Peak time (days)	Peak number of people	Peak time (days)	Peak number of people	Peak time (days)
New *E*	9.5 (5–11.5)	20	23 (16.5–25.5)	11	27.5 (23.5–30)	8
Accumulative *E*	147 (131–152)	30	160.5 (158–164)	18	163 (160–164.5)	12
New *E* in queues	7.5 (2–11.5)	17	22.5 (15.5–24.5)	9	27.5 (23.5–29.5)	8
Accumulative *E* in queues	117 (130.5–138.5)	30	152 (150–153.5)	18	153 (155.5–157.5)	12
New infectors	8 (7–11)	23	20 (16–24)	13	23.5 (19.5–25)	10
Accumulative infectors	140.5 (106.5–147.5)	30	160.5 (158–164)	21	163 (160–164.5)	15
New patients under treatment	2.5 (1.5–7)	27	5 (4–6.5)	18	6 (3–7.5)	15
Accumulative patients under treatment	32.5 (19.5–37.5)	30	47 (44–51)	24	49.5 (44–53)	22

The number of people at the peak is presented as a median value, with a fluctuation range of 25–75%, based on 50 simulations. *E* represents infected individuals. Infectors include *I*_m_ and *I*_h_.

**Figure 5:**
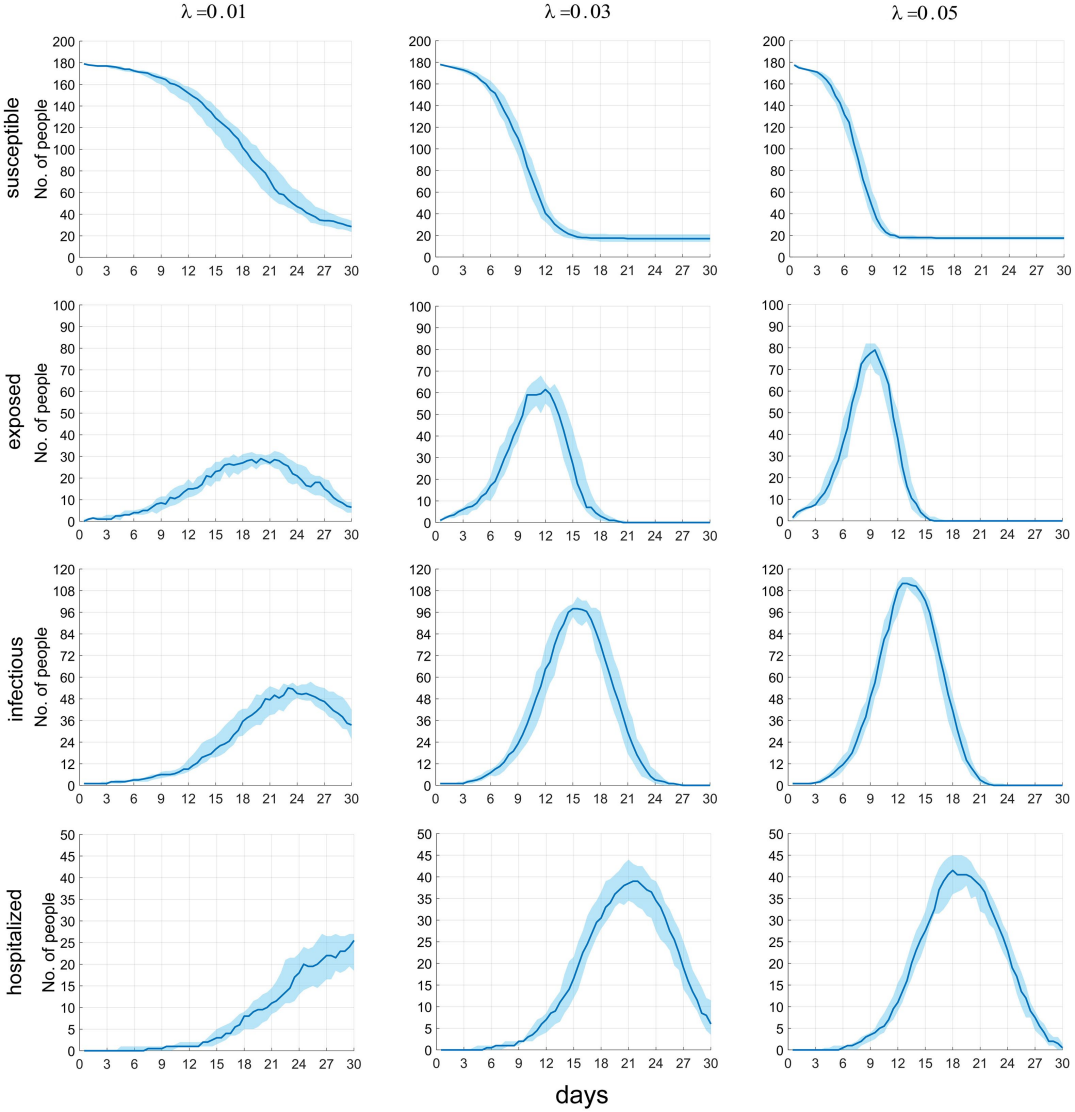
The time distribution of the current number of susceptible, latent infected, infectious and hospitalized individuals.

Figure 5 presents the temporal distribution of the current numbers of susceptible (*S*), exposed (*E*), infectious (including *I_m_* and *I_h_*) individuals, and patients under treatment (*H*). We can observe that as *λ* increases, the rate of decline in the number of *S* accelerates until all susceptible individuals are infected. The trends for the latter three compartments are largely similar, meaning that as *λ* increases, the growth rates of infected individuals and cases accelerate, the time to peak occurrence advances, and the peak values increase. Specific numerical values are provided in Table 3.

**Table 3:** The temporal distribution of the current number of groups in different infection states.

Epidemic-relatedStatistics	λ = 0.01		λ = 0.03		λ = 0.05	
	Peak number of people	Peak time (days)	Peak number of people	Peak time (days)	Peak number of people	Peak time (days)
Susceptible individuals (at the 7th day)	170.5 (166.5–172)	0	135 (125–149)	0	90 (78–108)	0
Latent infected individuals	29 (26–31)	20	61.5 (55–64.5)	12	79 (68.5–82)	9.5
Infectors	54 (44.5–54.5)	23	98 (90–104.5)	15	112 (110–115.5)	13
Patients under treatment	25.5 (18.5–27)	30	39 (33–42.5)	22	41.4 (36–45)	18

### 3.3 Sensitivity analyses

**Figure 6:**
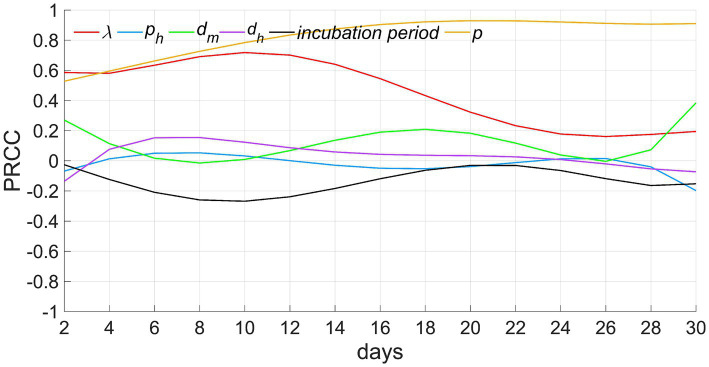
Results of sensitivity analyses. *λ* denotes the exposure index, *p_h_* signifies the rate of severe cases, *d_m_* depicts the infectious period for mild cases (from the onset of illness to recovery), *d_h_* indicates the infectious period for severe cases (from onset to treatment), and *p* represents the ratio of susceptible individuals to the total population when *t* equals 0.

In this study, we carried out sensitivity analyses on the model, utilizing six parameters and a continuous time-series regarding the overall cumulative number of infected individuals. A total of *N* = 50 samples were considered, procured from a uniform distribution across each parameter’s plausible range. The Partial Rank Correlation Coefficients (PRCCs) of these parameters exhibit a range from −1 to 1. PRCCs nearing −1 or 1 signal that the corresponding parameter exerts a significantly negative or positive impact on the output. Conversely, a value approximating 0 directs towards a lesser degree of output result influence from the respective parameter (as shown in Figure 6).

Among these parameters, (1) the proportion of susceptible individuals *p* demonstrates a consistent maximum positive correlation with the number of infected individuals, indicating that the larger the value of *p*, the greater the number of infected individuals. The correlation coefficient incrementally rises and maintains proximity to 1 throughout the time. This phenomena can be explained by the fact that the number of infected individuals gradually escalates in the initial stages of the epidemic. At a specific time, *t_0_*, the infected count reaches its apex, equivalent to the total count of all susceptible individuals, and ceases to elevate further. This limit is directly contingent upon the value of *p*. Therefore, the correlation between *p* and the count of infected individuals gradually strengthens before *t_0_* and persists with a strong positive interdependence, approaching 1, post *t_0_*. (2) The correlation of the exposure index *λ* exhibits a trend of initially increasing and then gradually decreasing. This is because, before time *t_0_*, an increase in *λ* leads to a higher probability of infection and a greater number of infected individuals. However, subsequent to *t_0_*, even if *λ* increases, the count of infected individuals has already attained its upper limit, prohibiting further escalation. Consequently, this circumstance leads to a diminishing positive correlation. (3) The infectious periods of mild cases *d_m_* and severe cases *d_h_* show a weak positive correlation with the number of infected individuals. This indicates that although an extension of the infectious period can increase the number of secondary infections, this effect is weaker compared to that of *p* and *λ*. (4) The correlation coefficient for the rate of severe cases *p_h_*, remains consistently close to 0, indicating that this parameter has a limited impact on the number of infected individuals. This reflects that the number of infections caused by mild cases and severe cases is similar. (5) The correlation coefficient of the incubation period is negative, and first descends and then ascends, indicating that the longer the incubation period, the slower the increase speed of infected individuals in the early stage of disease transmission. In the later stage, as the cumulative number of infected individuals approaches the upper limit, the negative correlation will gradually weaken.

## 4 Discussion

### 4.1 Innovation of the study

The innovation of this study is primarily manifested in the following two aspects: (1) Distinct research perspective. The transmission of infectious diseases while queuing for food represents a prevalent yet often overlooked human behavior. Using this as a starting point, we explore the transmission mechanism of diseases during the processes of queuing for food and dining, simulating the epidemic process of infectious diseases in everyday life scenarios. This constitutes an interesting and closely real-life-related research topic, further enriching the scope of theoretical epidemiology. (2) Innovative research method. Although we have previously developed several computer models to infer the epidemic progression of infectious diseases within populations, numerous challenges persist when implementing this method in research endeavors. This is evidenced by the following factors: First, the time and distance of contact between individuals during the food pick-up process are dynamically changing. These factors are vital for model establishment, but it is somewhat challenging to accurately determine them. Second, prior research regarding the determination of infection probability based on the time and distance of contact between susceptible individuals and the infection source is scarcely reported. Thus, there is a necessity to explore the establishment of a novel quantitative relationship. Practical experience has demonstrated that computer models possess significant flexibility and practicality. When designed appropriately, they can effectively resolve complex issues.

### 4.2 Variations in the number of individuals in the food pick-up queue

Simulating the dynamic food pick-up process presents a significant challenge for the model. To incorporate the time and location factors during the pick-up activity, we utilize a state data frame to record the information. Individuals in the food pick-up queue are segregated into those who are in the phase of picking up food and those who are waiting. The first column of the data frame logs the moment of position change for a food-picking individual. Position shifts among food-picking individuals may trigger accompanying changes among those in the waiting category. These waiting individuals, following their position changes, may either reach new food pick-up points or persist in waiting at newly adjusted spots, calling for further analysis and discernment. Once we calculate the fresh positions of food-picking individuals and potential new locations that the waiting ones may reach, we store the positional information in columns 2–11 of the data frame. Consequently, each row in the data frame chronicles a moment of position change for a food-picking person, as well as the subsequent positions of food-picking and waiting individuals after the queue readjusts. Upon the archiving of relevant information of all food-picking individuals, all close contacts of a single infection source in the identical queue and their contact timings can be discovered through referencing the time and position logs in the data frame.

Figure 2d shows a stable number of individuals involved in food pickup, reflecting a dynamic balance between those leaving the queue after pickup and those entering the area to start pickup. Such a phenomenon may be attributed to our implemented constraints on individual food pickup durations and quantities, averting queuing congestion instigated by an excessively prolonged food collection duration. Concurrently, hasty food collection did not enhance individual mobility or increase the number of food collectors. It required approximately seven minutes to retrieve food for 180 individuals from two different queues; a duration that closely mirrors common scenarios and validates our reasonable food retrieval timing parameters.

### 4.3 Formulation of the infection probability equation

Simulating the spread of disease requires calculating the probability of infection for close contacts, and then using this probability as a parameter to generate a Bernoulli random number (1 or 0) to determine whether each contact is infected or not. Accurately elucidating the correlation between infection probability, contact duration, and distance is an integral facet of model formulation. A review of existing literature reveals a conspicuous gap in this field, with the Wells-Riley infection model representing one of the few pertinent research findings (as shown in Equation 2) (19).


(2) P=1−exp(−Iqpt/Q)

In this context, *P* represents the probability of infection, *I* signifies the number of infection sources, *q* represents the generation rate of droplets and aerosols (the number of particles produced per hour), *p* is the pulmonary ventilation rate of a susceptible individual (cubic meters per second), *t* encapsulates exposure time (hours), and *Q* stands for the room ventilation rate (cubic meters per second). However, the essential parameters *q*, *p*, and *Q* within the equation pose inherent challenges in terms of measurement and prediction, and the variable for distance *d* is not included. As a result, there was a compelling need to revise the equation to align it with the research objectives. Based on practical experience and previous research results, we integrated the *d* variable into the equation, consolidated the parameters into a lambda *λ*, predicted its potential range and ultimately validated the robustness of the equation via a graphical representation. By evaluating Figures 2a–c and the primary results of the model, the formulation of Equation 1 is confirmed to be fundamentally sound.

### 4.4 Time distribution of infected individuals

From the first two rows of Figure 4, it can be observed that the majority of infections occur during the process of queuing for food (including waiting and picking-up food), likely due to the increased exposure to the infection source, with one infection source potentially affecting as many as 15 individuals in two queues. Dynamic contact further broadens the transmission scope within the population. In contrast, during dining, there are usually four people per table, seated fixedly with no inter-table disease transmission, thus limiting the contact scope. Fewer interactions with the infection source limits disease spread. This study implies that queues for food constitute a significant transmission risk during a respiratory disease outbreak. Actions to decrease queuing frequency, individual intake, and airborne pathogen density are recommended. Furthermore, the generally decreasing trend of *Rt* highlights that, as the number of susceptible individuals falls, so does the infection source’s transmissibility.

### 4.5 Limitations of the study

The limitations of the study are mainly reflected in four aspects. (1) For the convenience of research, we abstract the process of collective dining into a simplified model, while simultaneously removing some secondary behavioral factors from this process, such as conversation, air circulation, and multiple trips to get food. However, this idealized situation does not exist in reality, which not only leads to a certain degree of deviation between the results of the model and the actual situation, but also makes it difficult to evaluate the scientificity and rationality of the model by fitting the actual survey data with the model results. (2) Some parameter values were set by the authors based on their daily work and research experience. Additionally, the infection probability equation was also derived by the authors based on literature review. These assumptions made through subjective judgment rather than on-site investigations or scientific experiments can affect the accuracy of the prediction results. Therefore, the model still requires more precise data support from subsequent on-site investigations and laboratory tests, and further improvements are needed. (3) In order to targetedly analyze the dynamic mechanism of diseases transmission by queuing for food, we intentionally avoided other occasions of diseases transmission in the military, such as rest in the dormitory, group learning, and outdoor training. This simplification is not in line with the actual situation. However, if too many factors are added to the model, it will interfere with the research topic and make the calculation results difficult to interpret. (4) Compared with differential equation models, computer models have higher design difficulty and longer program runtime. As human activities become more complex, the difficulty of programming often increases exponentially, and it can lead to a decrease in computational efficiency and an extension of runtime. Therefore, this model may not necessarily be suitable for all research topics, and appropriate research methods should be selected based on the specific situation.

## 5 Conclusion

We have devised a computer model that uses an individual-based framework to simulate the dining experience of military personnel in a restaurant setting, thereby examining the transmission mechanism of respiratory infectious diseases. This novel model transcends the constraints of conventional dynamic models by accurately representing shifts in the contact duration and distance between queuing individuals, thereby depicting the disease transmission process in relation to population activity dynamics. The implementation of this model speaks to the precision and pragmatism of the research approach, while also broadening the realm of infectious disease dynamic studies. Our research identifies group queuing for food among soldiers as a pivotal element in disease transmission. In the absence of preventative and control measures, the disease will persistently proliferate among the population until all susceptible individuals become infected. For effective epidemic control, measures should be implemented during the epidemic phase. These measures may include dining in staggered periods, enhanced ventilation, and mandatory mask use during food collection.

## Data Availability

The datasets presented in this study can be found in online repositories. The names of the repository/repositories and accession number(s) can be found in the article/Supplementary material.
