# Supplementary material for: A theoretical epidemiological investigation into the transmission of respiratory infectious diseases during group meals among military personnel based on an individual-based model
Source: Front Public Health. 2025 May 21;13:1545938. doi: 10.3389/fpubh.2025.1545938 (PMC12133872; doi:10.3389/fpubh.2025.1545938)
Supplement: Supplementary file 1 [file Table_1.docx]

clear all

n_class=15; % Number of classes

m=12; % Number of students in each class

m_dishes=10; %the number of dishes

d1=1; % Distance from the person facing you during meals (meters)

d2=0.5; % The distance between two adjacent people in the same queue (meters)

d3=0.8; % Distance from the same side during meals

d4=0.8; % Distance from the person facing you during meals

lambda=0.03; %1-exp(-lambda*t/d^2), the floating range is 0.03-0.04, the unit of t is minutes

p_hos=0.29; % Hospitalization rate

d_syms=6; % Median symptom period is 6-7days

d_hos=9; % The hospitalization period is 9-11days

d_syms_hos=3.58; % Average number of days from symptoms to hospitalization

std_syms_hos=2.22; % Standard deviation from symptoms to hospitalization

t_b=7.5; % Breakfast time is 7:00

t_l=11.5; % Lunch time is 11:30

t_d=17.5; % Dinner time is 17:30

cycle=20; % Number of repeated cycles

T=30; % Number of days of epidemic transmission

p_inf=0.9; % Morbidity

mu_inc=3.1; sd_inc=2.6; %The incubation period

D_inf=[]; % Store the number of newly infected individuals per major cycle

D_inf_line=[]; % Store the number of new infected individuals per major cycle (ranks)

D_sick=[]; % Store the number of newly added patients for each major cycle

D_hos=[]; % Store the newly infected individuals generated during each large cycle of cooking

for i=1:cycle

pops=[1:n_class*m];

% IDs of personnel who will not be infected

no_inf0=binornd(1,1-p_inf,1,n_class*m);

no_inf=find(no_inf0==1);

susceptibles=pops;

susceptibles(no_inf)=[];

% Disorderly ordering of meals with fixed seats

Seats=[];

for j=1:n_class*m/4

Seats(j,:)=[(j-1)*4+1:j*4];

end

% Set infected person number 0 (as susceptible) when t=0

index0=round(rand*length(susceptibles)+0.5);

inf0=susceptibles(index0);

susceptibles(index0)=[];

ids_inf=[];

ids_inf(1)=inf0;

p_hos0=binornd(1,p_hos);

D=[];

% Store infected person information, with each column as follows: 1. Source of infection; 2 infected individuals; 3 classes; 4 incubation period;

% 5. Whether to be hospitalized; 6 infection time; 7. The time when the infection began; 8 Admission time (end of infection); 9 Discharge time

D(1,1)=0;D(1,2)=inf0; D(1,3)=ceil(D(1,2)/m); D(1,4)=0; D(1,5)=p_hos0;D(1,7)=0;

% infection period

if p_hos0==1

inf_peri=normrnd(d_syms_hos,std_syms_hos);

while inf_peri<1 | inf_peri>7

inf_peri=normrnd(d_syms_hos,std_syms_hos);

end

D(1,8)=D(1,7)+inf_peri;

D(1,9)=D(1,8)+d_hos+rand*2;

else

inf_peri=d_syms+rand;

D(1,8)=D(1,7)+inf_peri;

D(1,9)=0;

end

% Start small cycle for 1-T days

for j=1:T

[i,j]

for td=1:3

% Breakfast 7:30, Lunch 11:30, Dinner 17:30

if td==1

t=j-1+7.5/24;

elseif td==2

t=j-1+11.5/24;

elseif td==3

t=j-1+17.5/24;

end

% Find the person in place

ids_hos=D(find(D(:,5)==1 & D(:,8)<t & t<D(:,9)),2);

if length(ids_hos)>0

for iid=1:length(ids_hos)

fp=find(pops==ids_hos(iid));

pops(fp)=0;

end

pops(find(pops==0))=[];

end

% Meal time per person 10-15 minutes

time=rand(1,length(pops))*(15-10)+10;

Time=[time;pops];

% Find susceptible individuals in place

susceptibles=intersect(pops,susceptibles);

% Set the time for everyone to have breakfast

T_b=[]; % Store the meal time, and the first column represents the IDs of the person in attendance;

%The 2nd to 11th columns indicate the meal time; Each person plays 4-8 dishes, with each dish lasting for 3-8 seconds

T_b(:,1)=pops';

m_b=round(rand(1,length(pops))*(8-4+1)+0.5+3);

for k=1:length(pops)

nk=randperm(m_dishes);

nk=nk(1:m_b(k));

tk=rand(1,m_b(k))*(8-3+1)+0.5+2;

T_b(k,nk+1)=tk/60/60;

end

% Find all sources of infection at time t

rt_D=find(D(:,7)<t & t<D(:,8));

ids_inf=D(rt_D,2);

if length(ids_inf)>0

% Lines represents the queue sequence of all active personnel

ran=randperm(length(pops));

lines=pops(ran);

line1=lines(1:round(length(lines)/2));

line2=lines(length(line1)+1:length(lines));

State1=[]; State2=[];

for l=1:2

if l==1

line=line1;

else

line=line2;

end

% Establish a sequence of teams in chronological order, column 1: time; Columns 2-11:

% Person ID corresponding to each dish

% Initial order at time t

% Set the position of the first person in the meal sequence

r1=find(T_b(:,1)==line(1));

dishes=find(T_b(r1, 2:11)>0);

state0=zeros(1,10);

max_dish=max(dishes);

state0(max_dish)=line(1);

line(1)=[];

state=zeros(1,10);

state(max_dish)=1;

% Continue with the subsequent meal delivery team until someone arrives

% at the 10th position for meal delivery (queuing)

while state0(10)==0

% Find the next person's position in state0

r1=find(T_b(:,1)==line(1));

dishes=find(T_b(r1, 2:11)>0);

if max(dishes)<=max_dish

state0(max_dish+1)=line(1);

state(max_dish+1)=2;

max_dish=max_dish+1;

else

state0(max(dishes))=line(1);

state(max(dishes))=1;

max_dish=max(dishes);

end

line(1)=[];

end

state1=[t,state0];

if l==1

State1=[State1;state1];

else

State2=[State2;state1];

end

% Find the current cooking times of all the diners in their respective positions

f1=find(state==1);

id1=state0(f1);

r1=[];

for r=1:length(id1)

r1=[r1,find(T_b(:,1)==id1(r))];

end

t1=[];

f_next=[];

for k=1:length(r1)

t1(k)=T_b(r1(k),f1(k)+1);

% Next table for cooking

f3=find(T_b(r1(k),2:11)>0);

r3=find(f3==f1(k));

r3=r3-1;

if r3>0

f_next(k)=f3(r3);

else

f_next(k)=0;

end

end

% Next time change

while sum(state0>0)>0

move=0;

% Fill in Statei and add a new line for each change in the number of diners. Statei has 11 elements per

% row, with the first column representing the time and the second to eleventh columns representing

% the ids corresponding to the position

min_t=min(t1);

t1=t1-min_t;

rank_mint=find(t1==min(t1));

id0=id1(rank_mint);

rs=f1(rank_mint);

f_new=f_next(rank_mint);

% Determine if the person needs to move forward

if f_new==0

state0(rs)=0;

state(rs)=0;

% Remove it in f1, id1, t1, f_ Next

f1(rank_mint)=[];

id1(rank_mint)=[];

t1(rank_mint)=[];

f_next(rank_mint)=[];

move=2;

else

% Determine if the person can reach the next meal point

f_s0=find(state0(f_new:f1(rank_mint)-1)>0);

if length(f_s0)>0

fm=max(f_s0)+f_new-1;

f1(rank_mint)=[];

id1(rank_mint)=[];

t1(rank_mint)=[];

f_next(rank_mint)=[];

if 1<rs-fm

state0(rs)=0;

state0(fm+1)=id0;

state(rs)=0;

state(fm+1)=2;

move=1;

else

state(rs)=2;

end

else

state0(rs)=0;

state0(f_new)=id0;

state(rs)=0;

state(f_new)=1;

% Renew f1

f1(rank_mint)=f_new;

% Renew t1

ri=find(T_b(:,1)==id0);

t_new=T_b(ri,f_new+1);

t1(rank_mint)=t_new;

% Renew f_next

f2_11=find(T_b(ri,2:11)>0);

r11=find(f2_11==f_new);

if r11-1>0

f_next(rank_mint)=f2_11(r11-1);

else

f_next(rank_mint)=0;

end

move=1;

end

end

% When the person completes their movement, determine if there are any people waiting for meals

% but have not yet reached the designated location behind them, denoted by r0

if move==1 | move==2

r0=0;

if rs<length(state)

% Determine if there are any diners after this person

finds1=find(state(rs+1:length(state))==1);

if length(finds1)>0

ms=rs+min(finds1);

else

ms=length(state);

end

fs2=find(state(rs+1:ms)==2);

if length(fs2)>0

r0=length(fs2);

pos2=rs+fs2;

id2=state0(pos2);

end

end

if r0>0

% Determine the farthest reachable position of the first waiting person, represented by position1

fpm=find(state0(1:pos2(1)-1)>0);

if length(fpm)>0

position1=max(fpm)+1;

else

position1=1;

end

% Analysis of each waiter

for k=1:r0

%Find the next meal location for the waiter r_next

rk0=find(T_b(:,1)==id2(k));

frk0=find(T_b(rk0,2:11)>0);

r_next=frk0(max(find(frk0<pos2(k))));

if position1<=r_next

position_new=r_next;

position1=position_new+1;

% Renew state0, state

state0(pos2(k))=0;

state0(position_new)=id2(k);

state(pos2(k))=0;

state(position_new)=1;

% Renew f1, id1, t1, f_next

f1=find(state==1);

id1=state0(f1);

% Find the person in the position_ new arrival position of new (update f_next)

fid2=find(T_b(:,1)==id2(k));

f_id2=find(T_b(fid2,2:11)>0);

f_new=find(f_id2==position_new);

t_new=T_b(fid2,position_new+1);

f_new=f_new-1;

fs1_new=find(f1==position_new);

if fs1_new==1

t1=[t_new,t1];

if f_new>0

f_next=[f_id2(f_new),f_next];

else

f_next=[0,f_next];

end

elseif fs1_new==length(f1)

t1=[t1,t_new];

if f_new>0

f_next=[f_next,f_id2(f_new)];

else

f_next=[f_next,0];

end

elseif 1< fs1_new & fs1_new<length(f1)

t11=t1(1:fs1_new-1);

t12=t1(fs1_new:length(t1));

t1=[t11,t_new,t12];

f_next1=f_next(1:fs1_new-1);

f_next2=f_next(fs1_new:length(f_next));

if f_new>0

f_next=[f_next1,f_id2(f_new),f_next2];

else

f_next=[f_next1,0,f_next2];

end

end

else

position_new=position1;

position1=position_new+1;

if position_new<pos2(k)

state0(pos2(k))=0;

state0(position_new)=id2(k);

state(pos2(k))=0;

state(position_new)=2;

end

end

end

end

end

% When state0 (10) is 0, a new diner needs to be added

while state0(10)==0 & length(line)>0

fT=find(T_b(:,1)==line(1));

md=max(find(T_b(fT,2:11)>0));

md0=max(find(state0>0));

if md<=md0

state0(md0+1)=line(1);

state(md0+1)=2;

else

state0(md)=line(1);

state(md)=1;

f1=[f1,md];

id1=[id1,line(1)];

t_new=T_b(fT,md+1);

t1=[t1,t_new];

% Renew f_next

f_fline1=find(T_b(fT,2:11)>0);

f_new=find(f_fline1==md);

f_new=f_new-1;

f_next=[f_next,f_fline1(f_new)];

end

line(1)=[];

end

% Continue to Statei

if l==1 & move>0

[r,c]=size(State1);

state1=[State1(r,1)+min_t,state0];

State1=[State1;state1];

elseif l==2 & move>0

[r,c]=size(State2);

state1=[State2(r,1)+min_t,state0];

State2=[State2;state1];

end

end

end

% The Statei for breakfast/lunch/dinner has been completed

% Find the transmission relationship of each infectious agent in the queue

for k=1: length(ids_inf)

% Find the close contacts on the same line first

% First, determine which line the source of infection is in

f_line1=find(line1==ids_inf(k));

if length(f_line1)>0

line=line1;

line_another=line2;

State=State1;

State_another=State2;

else

line=line2;

line_another=line1;

State=State2;

State_another=State1;

end

[rS,cS]=size(State);

State(rS,:)=[];

[rS,cS]=size(State_another);

State_another(rS,:)=[];

% Storing the time during the movement of infectious agents in the line (including meals and standby)

% Any element of the line that moves is counted, and the time of change is used to

% calculate the close connection of the line

line0=[State(1,2:11),line(find(line==State(1,11))+1:length(line))];

r_inf=find(line0==ids_inf(k));

% The close contact of the source of infection in the bank

% When the infectious source is in the line, the time when the position of any person changes is

% used to calculate all close contacts of the infectious source in the line

[ri,ci]=find(State(:,2:11)==ids_inf(k));

r_line=1:max(ri);

time_line=State(r_line,1);

If=[time_line,ones(length(r_line),1)*5];

% The close contact of the source of infection on another line

% The source of infection is on line_ The time when the position changes in behind

if r_inf>11

% The source of infection is on line_ Position change in behind

rfi=find(State(:,11)==ids_inf(k));

rmin=min(rfi);

[ru_b,cu_b]=unique(State(1:rmin-1,1),'stable');

[ru_l,cu_l]=unique(State(rmin:max(ri),1),'stable');

time_line=[ru_b;ru_l];

Ia=[time_line,ones(length(time_line),1)];

else

[ru_l,cu_l]=unique(State(ri,1),'stable');

Ia=[ru_l,ones(length(ru_l),1)];

end

% Find line_ Time when another moves

line_an=find(State_another(:,1)<=State(max(ri),1));

time_line=State_another(line_an,1);

Ib=[time_line,ones(length(time_line),1)*2];

If=[If;Ia;Ib];

If=sortrows(If,1)';

% Calculate the close connection between the infectious source on another line and this line,

% and store it in Contacts_ In ni

% Calculate each column in If, where each column represents a time point of change

Contacts_ni=[];

for ni=1:length(If(1,:))

% Establish line_ni

r_ni=State(:,1)==If(1,ni);

if State(r_ni,11)>0

f0=find(line==State(r_ni,11));

if f0+1<=length(line)

line_ni_behind=line(f0+1:length(line));

line_ni=[State(r_ni,2:11),line_ni_behind];

else

line_ni=State(r_ni,2:11);

end

else

line_ni=State(r_ni,2:11);

end

% Determine r_ Line_ ni, i.e. the rank of infection is on line_ ni

r_line_ni=find(line_ni==ids_inf(k));

if If(2,ni)==5

distances=[-4:-1,1:4];

line_t=line_ni;

else

% The rank established at If(1,ni) of line_ni_an

r_ni_an=max(find(State_another(:,1)<=If(1,ni)));

if State_another(r_ni_an,11)>0

f0=find(line_another==State_another(r_ni_an,11));

if f0+1<=length(line_another)

line_behind_an=line_another(f0+1:length(line_another));

line_ni_an=[State_another(r_ni_an,2:11),line_behind_an];

else

line_ni_an=State_another(r_ni_an,2:11);

end

else

line_ni_an=State_another(r_ni_an,2:11);

end

distances=[-3:-1,1:3];

line_t=line_ni_an;

end

% Determine the connections in line_ t

for nj=1:length(distances)

r_ni=r_line_ni+distances(nj);

if 1<=r_ni & r_ni<=length(line_t) & line_t(r_ni)>0

contacts_ni(1)=line_t(r_ni);

contacts_ni(2)=distances(nj);

contacts_ni(3)=If(1,ni);

Contacts_ni=[Contacts_ni,contacts_ni'];

end

end

end

Contacters=[];

c_ni=length(Contacts_ni(1,:));

while c_ni>0

id_con=Contacts_ni(1,1);

distance=Contacts_ni(2,1);

fids=find(Contacts_ni(1,:)==id_con);

t_min=min(Contacts_ni(3,fids));

t_last=max(Contacts_ni(3,fids));

contacters0=[id_con,distance,t_min,t_last-t_min]';

Contacters=[Contacters,contacters0];

Contacts_ni(:,fids)=[];

c_ni=length(Contacts_ni(1,:));

end

[elements, rC, rs]=intersect(Contacters(1,:), susceptibles);

Contacters=Contacters(:,rC);

% Calculate the transmission relationship of this infectious source during standby

% and meal preparation processes as follows

for ni=1:length(Contacters(1,:))

t_ni=Contacters(4,ni);

d_ni=Contacters(2,ni);

par_ni=1-exp(-lambda*t_ni*60/d_ni^2);

p_ni=binornd(1,par_ni);

if p_ni==1

% Add the propagation relationship to D

% Each column is: 1 Source of infection; 2 Infected individuals; 3 classes of infected individuals;

% 4 incubation period; 5. Whether to be hospitalized; 6 infection time; 7. Starting time of infection;

% 8 Admission time; 9. Discharge time; 10 infection locations (1 dozen meals and 2 meals)

% The current number of rows in D

[rD,cD]=size(D);

rD=rD+1;

D(rD,1)=ids_inf(k); D(rD,2)=Contacters(1,ni); D(rD,3)=ceil(D(rD,2)/m);

inc_per=normrnd(mu_inc, sd_inc);

while inc_per<1 | inc_per>5

inc_per=normrnd(mu_inc, sd_inc);

end

D(rD,4)=inc_per;

D(rD,5)=binornd(1,p_hos);

D(rD,6)=Contacters(3,ni)+rand*Contacters(4,ni);

D(rD,7)=D(rD,6)+D(rD,4);

% infection period

if D(rD,5)==1

inf_peri=normrnd(d_syms_hos,std_syms_hos);

while inf_peri<1 | inf_peri>7

inf_peri=normrnd(d_syms_hos,std_syms_hos);

end

D(rD,8)=D(rD,7)+inf_peri;

D(rD,9)=D(rD,8)+d_hos+rand*(11-d_hos);

else

D(rD,8)=D(rD,7)+rand+6;

D(rD,9)=0;

end

susceptibles(find(susceptibles==D(rD,2)))=[];

D(rD,10)=1;

end

end

end

% Calculate the propagation relationship during meals

% Find the seat with the source of infection first, source of infection ids_ inf, Pops for current personnel, Seats for seats

Inf=zeros(length(Seats(:,1)),4);

% Identify the source of infection

for is=1:length(ids_inf)

[rS,cS]=find(Seats==ids_inf(is));

r0=sum(find(Inf(rS,:)>0));

Inf(rS,r0+1)=ids_inf(is);

end

% Identify susceptible individuals

infected=[D(:,2)',no_inf];

Seats0=Seats;

for in=1:length(infected)

[rS,cS]=find(Seats0==infected(in));

Seats0(rS,cS)=0;

end

Sus=Seats0;

% Remove rows without susceptible individuals

f0=find(sum(Sus>0,2)==0);

Sus(f0,:)=[];

Inf(f0,:)=[];

% Remove rows without any source of infection

f0=find(sum(Inf>0,2)==0);

Inf(f0,:)=[];

Sus(f0,:)=[];

% Calculate the spread of the epidemic at each dining table

for is=1:length(Inf(:,1))

sus_is=Sus(is,find(Sus(is,:)>0));

inf_is=Inf(is,find(Inf(is,:)>0));

si=[sus_is,inf_is];

t_max=[];

for it=1:length(si)

t_max(it)=Time(1,find(Time(2,:)==si(it)));

end

t_max=max(t_max);

for mi=3:length(sus_is)

% The probability of susceptible individuals being infected by a single source of infection

pt=1-(1-pt)^length(inf_is);

pt=binornd(1,pt);

if pt==1

susceptibles(find(susceptibles==sus_is(mi)))=[];

% Determine which source of infection is causing the infection

inf_source=inf_is(round(rand*length(inf_is)+0.5));

[rD,cD]=size(D);

rD=rD+1;

D(rD,1)=inf_source; D(rD,2)=sus_is(mi); D(rD,3)=ceil(D(rD,2)/m);

inc_per=normrnd(mu_inc, sd_inc);

while inc_per<1 | inc_per>5

inc_per=normrnd(mu_inc, sd_inc);

end

D(rD,4)=inc_per;

D(rD,5)=binornd(1,p_hos);

%Infection time D(rD,6)

% Time when the infectious agent left the queue

[ri,ci]=find(State(:,2:11)==D(rD,1));

if length(ri)>0

t_inf_arr=State(max(ri),1);

else

[ri,ci]=find(State_another(:,2:11)==D(rD,1));

t_inf_arr=State_another(max(ri),1);

end

% The time when susceptible individuals leave the queue

[ri,ci]=find(State(:,2:11)==D(rD,2));

if length(ri)>0

t_sus_arr=State(max(ri),1);

else

[ri,ci]=find(State_another(:,2:11)==D(rD,2));

t_sus_arr=State_another(max(ri),1);

end

t0=max(t_inf_arr,t_sus_arr);

D(rD,6)=t0+rand*t_max/60;

D(rD,7)=D(rD,6)+D(rD,4);

% infection period

if D(rD,5)==1

inf_peri=normrnd(d_syms_hos,std_syms_hos);

while inf_peri<1 | inf_peri>7

inf_peri=normrnd(d_syms_hos,std_syms_hos);

end

D(rD,8)=D(rD,7)+inf_peri;

D(rD,9)=D(rD,8)+d_hos+rand*(11-d_hos);

else

D(rD,8)=D(rD,7)+6+rand;

D(rD,9)=0;

end

D(rD,10)=2;

end

end

end

end

end

end

% Count the new infected individuals added on the day of the i-th cycle

for day=1:T

D_inf(i,day)=length(find(day-1<=D(:,6) & D(:,6)<day));

D_inf_line(i,day)=length(find(day-1<=D(:,6) & D(:,6)<day & D(:,10)==1));

D_sick(i,day)=length(find(day-1<=D(:,7) & D(:,7)<day));

D_hos(i,day)=length(find(D(:,5)==1 & day-1<=D(:,8) & D(:,8)<day));

end

end

D_inf_cum=cumsum(D_inf,2);

D_line_cum=cumsum(D_inf_line,2);

D_sick_cum=cumsum(D_sick,2);

D_hos_cum=cumsum(D_hos,2);

%lambda=0.03, 0.04, 0.05

hold on

left=10;

right=60;

T=30;

t=1:30;

x=[t,flip(t)];

Inf_P75=prctile(D_hos,75);

Inf_P50=prctile(D_hos,50);

Inf_P25=prctile(D_hos,25);

E1=[Inf_P75,flip(Inf_P25)];

f1=fill(x, E1, 'b','facealpha',0.4,'edgealpha',0);

Cum_P75=prctile(D_hos_cum,75);

Cum_P50=prctile(D_hos_cum,50);

Cum_P25=prctile(D_hos_cum,25);

E2=[Cum_P75,flip(Cum_P25)];

f2=fill(x, E2*left/right, 'b','facealpha',0.4,'edgealpha',0);

% draw

[ax,h1,h2]=plotyy(t,Inf_P50,t,Cum_P50)

xlim([0,T])

set(ax(1),'ylim',[0,left])

set(ax(2),'ylim',[0,right])

set(ax,'Ytick',[])

set(ax(1),'Ytick',[0:left/10:left])

set(ax(2),'Ytick',[0:right/10:right])

set(h1,'LineStyle','-','LineWidth',2,'color','b');

set(h2,'LineStyle','--','LineWidth',2,'color','b');

set(ax(1),'fontsize',16,'ycolor','k')

set(ax(2),'fontsize',16,'ycolor','k')

HH1=get(ax(1),'Ylabel');

set(HH1,'String','No. of new hospitalized','fontname','arial','fontsize',20);

HH2=get(ax(2),'Ylabel');

%set(HH2,'String','No. of accumulative hospitalized','fontname','arial','fontsize',20);

set (gca,'position',[0.13,0.17,0.71,0.8] );

set(gca,'Xtick',[])

set(gca,'Xtick',[0:T/10:T]);

h=legend([h1,h2],'\fontsize{20}\itnew hospitalized','\fontsize{20}\itaccumulative hospitalized','\fontsize{20}','Location','NorthEast')

set(h,'box','off')

box on

grid on
