# Supplementary material for: A theoretical epidemiological investigation into the transmission of respiratory infectious diseases during group meals among military personnel based on an individual-based model
Source: Front Public Health. 2025 May 21;13:1545938. doi: 10.3389/fpubh.2025.1545938 (PMC12133872; doi:10.3389/fpubh.2025.1545938)
Supplement: Supplementary file 2 [file Table_2.docx]

clear all

n_class=15; % Number of classes

m=12; % Number of students in each class

m_dishes=10; %the number of dishes

d1=1; % Distance from the person facing you during meals (meters)

d2=0.5; % The distance between two adjacent people in the same queue (meters)

d3=0.8; % Distance from the same side during meals

d4=0.8; % Distance from the person facing you during meals

d_hos=9; % The hospitalization period is 9-11days

std_syms_hos=2.22; % Standard deviation from symptoms to hospitalization

t_b=7.5; % Breakfast time is 7:00

t_l=11.5; % Lunch time is 11:30

t_d=17.5; % Dinner time is 17:30

sd_inc=2.6; %The incubation period

ss=50;

cycle=50;

Ro=[];

for i=1:cycle

Ran_lambda=randsample([1:ss],ss);

lambda=0.03*0.5+(Ran_lambda-1)*0.03/ss+0.03/ss*rand;

Ran_p_hos=randsample([1:ss],ss);

p_hos=0.29*0.5+(Ran_p_hos-1)*0.29/ss+0.29/ss*rand;

Ran_d_syms=randsample([1:ss],ss);

d_syms=6*0.5+(Ran_d_syms-1)*6/ss+6/ss*rand;

Ran_d_syms_hos=randsample([1:ss],ss);

d_syms_hos=3.58*0.5+(Ran_d_syms_hos-1)*3.58/ss+3.58/ss*rand;

Ran_mu_inc=randsample([1:ss],ss);

mu_inc=3.1*0.5+(Ran_mu_inc-1)*3.1/ss+3.1/ss*rand;

Ran_p_inf=randsample([1:ss],ss);

p_inf=0.9*0.5+(Ran_p_inf-1)*0.9/ss+0.9/ss*rand;

Values=[];

Values(:,1)=lambda'; Values(:,2)=p_hos'; Values(:,3)=d_syms'; Values(:,4)=d_syms_hos'; Values(:,5)=mu_inc'; Values(:,6)=p_inf';

Ranks=[];

Ranks(:,1)=Ran_lambda'; Ranks(:,2)=Ran_p_hos'; Ranks(:,3)=Ran_d_syms'; Ranks(:,4)=Ran_d_syms_hos'; Ranks(:,5)=Ran_mu_inc'; Ranks(:,6)=Ran_p_inf';

results=[];

for j=1:ss

pops=[1:n_class*m];

no_inf0=binornd(1,1-Values(j,6),1,n_class*m);

no_inf=find(no_inf0==1);

susceptibles=pops;

susceptibles(no_inf)=[];

Seats=[];

for j0=1:n_class*m/4

Seats(j0,:)=[(j0-1)*4+1:j0*4];

end

index0=round(rand*length(susceptibles)+0.5);

inf0=susceptibles(index0);

susceptibles(index0)=[];

ids_inf=[];

ids_inf(1)=inf0;

p_hos0=binornd(1,Values(j,2));

D=[];

D(1,1)=0;D(1,2)=inf0; D(1,3)=ceil(D(1,2)/m); D(1,4)=0; D(1,5)=p_hos0;D(1,7)=0;

if p_hos0==1

inf_peri=normrnd(Values(j,4),std_syms_hos);

while inf_peri<1 | inf_peri>7

inf_peri=normrnd(Values(j,4),std_syms_hos);

end

D(1,8)=D(1,7)+inf_peri;

D(1,9)=D(1,8)+d_hos+rand*2;

else

inf_peri=Values(j,3)+rand;

D(1,8)=D(1,7)+inf_peri;

D(1,9)=0;

end

for jj=1:2*i

[i,j,jj]

for td=1:3

if td==1

t=jj-1+7.5/24;

elseif td==2

t=jj-1+11.5/24;

elseif td==3

t=jj-1+17.5/24;

end

ids_hos=D(find(D(:,5)==1 & D(:,8)<t & t<D(:,9)),2);

if length(ids_hos)>0

for iid=1:length(ids_hos)

fp=find(pops==ids_hos(iid));

pops(fp)=0;

end

pops(find(pops==0))=[];

end

time=rand(1,length(pops))*(15-10)+10;

Time=[time;pops];

susceptibles=intersect(pops,susceptibles);

T_b=[];

T_b(:,1)=pops';

m_b=round(rand(1,length(pops))*(8-4+1)+0.5+3);

for k=1:length(pops)

nk=randperm(m_dishes);

nk=nk(1:m_b(k));

tk=rand(1,m_b(k))*(8-3+1)+0.5+2;

T_b(k,nk+1)=tk/60/60;

end

rt_D=find(D(:,7)<t & t<D(:,8));

ids_inf=D(rt_D,2);

if length(ids_inf)>0

ran=randperm(length(pops));

lines=pops(ran);

line1=lines(1:round(length(lines)/2));

line2=lines(length(line1)+1:length(lines));

State1=[]; State2=[];

for l=1:2

if l==1

line=line1;

else

line=line2;

end

r1=find(T_b(:,1)==line(1));

dishes=find(T_b(r1, 2:11)>0);

state0=zeros(1,10);

max_dish=max(dishes);

state0(max_dish)=line(1);

line(1)=[];

state=zeros(1,10);

state(max_dish)=1;

while state0(10)==0

r1=find(T_b(:,1)==line(1));

dishes=find(T_b(r1, 2:11)>0);

if max(dishes)<=max_dish

state0(max_dish+1)=line(1);

state(max_dish+1)=2;

max_dish=max_dish+1;

else

state0(max(dishes))=line(1);

state(max(dishes))=1;

max_dish=max(dishes);

end

line(1)=[];

end

state1=[t,state0];

if l==1

State1=[State1;state1];

else

State2=[State2;state1];

end

f1=find(state==1);

id1=state0(f1);

r1=[];

for r=1:length(id1)

r1=[r1,find(T_b(:,1)==id1(r))];

end

t1=[];

f_next=[];

for k=1:length(r1)

t1(k)=T_b(r1(k),f1(k)+1);

f3=find(T_b(r1(k),2:11)>0);

r3=find(f3==f1(k));

r3=r3-1;

if r3>0

f_next(k)=f3(r3);

else

f_next(k)=0;

end

end

while sum(state0>0)>0

move=0;

min_t=min(t1);

t1=t1-min_t;

rank_mint=find(t1==min(t1));

id0=id1(rank_mint);

rs=f1(rank_mint);

f_new=f_next(rank_mint);

if f_new==0

state0(rs)=0;

state(rs)=0;

f1(rank_mint)=[];

id1(rank_mint)=[];

t1(rank_mint)=[];

f_next(rank_mint)=[];

move=2;

else

f_s0=find(state0(f_new:f1(rank_mint)-1)>0);

if length(f_s0)>0

fm=max(f_s0)+f_new-1;

f1(rank_mint)=[];

id1(rank_mint)=[];

t1(rank_mint)=[];

f_next(rank_mint)=[];

if 1<rs-fm

state0(rs)=0;

state0(fm+1)=id0;

state(rs)=0;

state(fm+1)=2;

move=1;

else

state(rs)=2;

end

else

state0(rs)=0;

state0(f_new)=id0;

state(rs)=0;

state(f_new)=1;

f1(rank_mint)=f_new;

ri=find(T_b(:,1)==id0);

t_new=T_b(ri,f_new+1);

t1(rank_mint)=t_new;

f2_11=find(T_b(ri,2:11)>0);

r11=find(f2_11==f_new);

if r11-1>0

f_next(rank_mint)=f2_11(r11-1);

else

f_next(rank_mint)=0;

end

move=1;

end

end

if move==1 | move==2

r0=0;

if rs<length(state)

finds1=find(state(rs+1:length(state))==1);

if length(finds1)>0

ms=rs+min(finds1);

else

ms=length(state);

end

fs2=find(state(rs+1:ms)==2);

if length(fs2)>0

r0=length(fs2);

pos2=rs+fs2;

id2=state0(pos2);

end

end

if r0>0

fpm=find(state0(1:pos2(1)-1)>0);

if length(fpm)>0

position1=max(fpm)+1;

else

position1=1;

end

for k=1:r0

rk0=find(T_b(:,1)==id2(k));

frk0=find(T_b(rk0,2:11)>0);

r_next=frk0(max(find(frk0<pos2(k))));

if position1<=r_next

position_new=r_next;

position1=position_new+1;

state0(pos2(k))=0;

state0(position_new)=id2(k);

state(pos2(k))=0;

state(position_new)=1;

f1=find(state==1);

id1=state0(f1);

fid2=find(T_b(:,1)==id2(k));

f_id2=find(T_b(fid2,2:11)>0);

f_new=find(f_id2==position_new);

t_new=T_b(fid2,position_new+1);

f_new=f_new-1;

fs1_new=find(f1==position_new);

if fs1_new==1

t1=[t_new,t1];

if f_new>0

f_next=[f_id2(f_new),f_next];

else

f_next=[0,f_next];

end

elseif fs1_new==length(f1)

t1=[t1,t_new];

if f_new>0

f_next=[f_next,f_id2(f_new)];

else

f_next=[f_next,0];

end

elseif 1< fs1_new & fs1_new<length(f1)

t11=t1(1:fs1_new-1);

t12=t1(fs1_new:length(t1));

t1=[t11,t_new,t12];

f_next1=f_next(1:fs1_new-1);

f_next2=f_next(fs1_new:length(f_next));

if f_new>0

f_next=[f_next1,f_id2(f_new),f_next2];

else

f_next=[f_next1,0,f_next2];

end

end

else

position_new=position1;

position1=position_new+1;

if position_new<pos2(k)

state0(pos2(k))=0;

state0(position_new)=id2(k);

state(pos2(k))=0;

state(position_new)=2;

end

end

end

end

end

while state0(10)==0 & length(line)>0

fT=find(T_b(:,1)==line(1));

md=max(find(T_b(fT,2:11)>0));

md0=max(find(state0>0));

if md<=md0

state0(md0+1)=line(1);

state(md0+1)=2;

else

state0(md)=line(1);

state(md)=1;

f1=[f1,md];

id1=[id1,line(1)];

t_new=T_b(fT,md+1);

t1=[t1,t_new];

f_fline1=find(T_b(fT,2:11)>0);

f_new=find(f_fline1==md);

f_new=f_new-1;

f_next=[f_next,f_fline1(f_new)];

end

line(1)=[];

end

if l==1 & move>0

[r,c]=size(State1);

state1=[State1(r,1)+min_t,state0];

State1=[State1;state1];

elseif l==2 & move>0

[r,c]=size(State2);

state1=[State2(r,1)+min_t,state0];

State2=[State2;state1];

end

end

end

for k=1: length(ids_inf)

f_line1=find(line1==ids_inf(k));

if length(f_line1)>0

line=line1;

line_another=line2;

State=State1;

State_another=State2;

else

line=line2;

line_another=line1;

State=State2;

State_another=State1;

end

[rS,cS]=size(State);

State(rS,:)=[];

[rS,cS]=size(State_another);

State_another(rS,:)=[];

line0=[State(1,2:11),line(find(line==State(1,11))+1:length(line))];

r_inf=find(line0==ids_inf(k));

[ri,ci]=find(State(:,2:11)==ids_inf(k));

r_line=1:max(ri);

time_line=State(r_line,1);

If=[time_line,ones(length(r_line),1)*5];

if r_inf>11

rfi=find(State(:,11)==ids_inf(k));

rmin=min(rfi);

[ru_b,cu_b]=unique(State(1:rmin-1,1),'stable');

[ru_l,cu_l]=unique(State(rmin:max(ri),1),'stable');

time_line=[ru_b;ru_l];

Ia=[time_line,ones(length(time_line),1)];

else

[ru_l,cu_l]=unique(State(ri,1),'stable');

Ia=[ru_l,ones(length(ru_l),1)];

end

line_an=find(State_another(:,1)<=State(max(ri),1));

time_line=State_another(line_an,1);

Ib=[time_line,ones(length(time_line),1)*2];

If=[If;Ia;Ib];

If=sortrows(If,1)';

Contacts_ni=[];

for ni=1:length(If(1,:))

r_ni=State(:,1)==If(1,ni);

if State(r_ni,11)>0

f0=find(line==State(r_ni,11));

if f0+1<=length(line)

line_ni_behind=line(f0+1:length(line));

line_ni=[State(r_ni,2:11),line_ni_behind];

else

line_ni=State(r_ni,2:11);

end

else

line_ni=State(r_ni,2:11);

end

r_line_ni=find(line_ni==ids_inf(k));

if If(2,ni)==5

distances=[-4:-1,1:4];

line_t=line_ni;

else

r_ni_an=max(find(State_another(:,1)<=If(1,ni)));

if State_another(r_ni_an,11)>0

f0=find(line_another==State_another(r_ni_an,11));

if f0+1<=length(line_another)

line_behind_an=line_another(f0+1:length(line_another));

line_ni_an=[State_another(r_ni_an,2:11),line_behind_an];

else

line_ni_an=State_another(r_ni_an,2:11);

end

else

line_ni_an=State_another(r_ni_an,2:11);

end

distances=[-3:-1,1:3];

line_t=line_ni_an;

end

for nj=1:length(distances)

r_ni=r_line_ni+distances(nj);

if 1<=r_ni & r_ni<=length(line_t) & line_t(r_ni)>0

contacts_ni(1)=line_t(r_ni);

contacts_ni(2)=distances(nj);

contacts_ni(3)=If(1,ni);

Contacts_ni=[Contacts_ni,contacts_ni'];

end

end

end

Contacters=[];

c_ni=length(Contacts_ni(1,:));

while c_ni>0

id_con=Contacts_ni(1,1);

distance=Contacts_ni(2,1);

fids=find(Contacts_ni(1,:)==id_con);

t_min=min(Contacts_ni(3,fids));

t_last=max(Contacts_ni(3,fids));

contacters0=[id_con,distance,t_min,t_last-t_min]';

Contacters=[Contacters,contacters0];

Contacts_ni(:,fids)=[];

c_ni=length(Contacts_ni(1,:));

end

[elements, rC, rs]=intersect(Contacters(1,:), susceptibles);

Contacters=Contacters(:,rC);

for ni=1:length(Contacters(1,:))

t_ni=Contacters(4,ni);

d_ni=Contacters(2,ni);

par_ni=1-exp(-Values(j,1)*t_ni*60/d_ni^2);

p_ni=binornd(1,par_ni);

if p_ni==1

[rD,cD]=size(D);

rD=rD+1;

D(rD,1)=ids_inf(k); D(rD,2)=Contacters(1,ni); D(rD,3)=ceil(D(rD,2)/m);

inc_per=normrnd(Values(j,5), sd_inc);

while inc_per<1 | inc_per>5

inc_per=normrnd(Values(j,5), sd_inc);

end

D(rD,4)=inc_per;

D(rD,5)=binornd(1,Values(j,2));

D(rD,6)=Contacters(3,ni)+rand*Contacters(4,ni);

D(rD,7)=D(rD,6)+D(rD,4);

if D(rD,5)==1

inf_peri=normrnd(Values(j,4),std_syms_hos);

while inf_peri<1 | inf_peri>7

inf_peri=normrnd(Values(j,4),std_syms_hos);

end

D(rD,8)=D(rD,7)+inf_peri;

D(rD,9)=D(rD,8)+d_hos+rand*(11-d_hos);

else

D(rD,8)=D(rD,7)+rand+Values(j,3);

D(rD,9)=0;

end

susceptibles(find(susceptibles==D(rD,2)))=[];

D(rD,10)=1;

end

end

end

Inf=zeros(length(Seats(:,1)),4);

for is=1:length(ids_inf)

[rS,cS]=find(Seats==ids_inf(is));

r0=sum(find(Inf(rS,:)>0));

Inf(rS,r0+1)=ids_inf(is);

end

infected=[D(:,2)',no_inf];

Seats0=Seats;

for in=1:length(infected)

[rS,cS]=find(Seats0==infected(in));

Seats0(rS,cS)=0;

end

Sus=Seats0;

f0=find(sum(Sus>0,2)==0);

Sus(f0,:)=[];

Inf(f0,:)=[];

f0=find(sum(Inf>0,2)==0);

Inf(f0,:)=[];

Sus(f0,:)=[];

for is=1:length(Inf(:,1))

sus_is=Sus(is,find(Sus(is,:)>0));

inf_is=Inf(is,find(Inf(is,:)>0));

si=[sus_is,inf_is];

t_max=[];

for it=1:length(si)

t_max(it)=Time(1,find(Time(2,:)==si(it)));

end

t_max=max(t_max);

for mi=3:length(sus_is)

pt=1-exp(-Values(j,1)*t_max/0.8^2);

pt=1-(1-pt)^length(inf_is);

pt=binornd(1,pt);

if pt==1

susceptibles(find(susceptibles==sus_is(mi)))=[];

inf_source=inf_is(round(rand*length(inf_is)+0.5));

[rD,cD]=size(D);

rD=rD+1;

D(rD,1)=inf_source; D(rD,2)=sus_is(mi); D(rD,3)=ceil(D(rD,2)/m);

inc_per=normrnd(Values(j,5), sd_inc);

while inc_per<1 | inc_per>5

inc_per=normrnd(Values(j,5), sd_inc);

end

D(rD,4)=inc_per;

D(rD,5)=binornd(1,Values(j,2));

[ri,ci]=find(State(:,2:11)==D(rD,1));

if length(ri)>0

t_inf_arr=State(max(ri),1);

else

[ri,ci]=find(State_another(:,2:11)==D(rD,1));

t_inf_arr=State_another(max(ri),1);

end

[ri,ci]=find(State(:,2:11)==D(rD,2));

if length(ri)>0

t_sus_arr=State(max(ri),1);

else

[ri,ci]=find(State_another(:,2:11)==D(rD,2));

t_sus_arr=State_another(max(ri),1);

end

t0=max(t_inf_arr,t_sus_arr);

D(rD,6)=t0+rand*t_max/60;

D(rD,7)=D(rD,6)+D(rD,4);

if D(rD,5)==1

inf_peri=normrnd(Values(j,4),std_syms_hos);

while inf_peri<1 | inf_peri>7

inf_peri=normrnd(Values(j,4),std_syms_hos);

end

D(rD,8)=D(rD,7)+inf_peri;

D(rD,9)=D(rD,8)+d_hos+rand*(11-d_hos);

else

D(rD,8)=D(rD,7)+Values(j,3)+rand;

D(rD,9)=0;

end

D(rD,10)=2;

end

end

end

end

end

end

results(j)=length(D(:,1));

end

[i1,i2]=sort(results);

[i3,i4]=sort(i2);

Ran_r=i4';

for r=1:6

XX1=[];

for iii=1:6

if iii~=r

XX1(:,iii)=Ranks(:,iii);

else

XX1(:,iii)=ones(length(results),1);

end

end

XX2=XX1*inv(XX1'*XX1)*XX1'*Ranks(:,r);

YY2=XX1*inv(XX1'*XX1)*XX1'*Ran_r;

Xz=Ranks(:,r)-XX2;

Yz=Ran_r-YY2;

Ro(i,r)=dot(Xz,Yz)/norm(Xz)/norm(Yz);

end

Ro

xlswrite('C:\Users\LENOVO\Desktop\Ro.xls',Ro)

end

Ro=xlsread('C:\Users\LENOVO\Desktop\Ro.xls');

hold on

t=[1:15];

k=5;

M1=Ro(:,1);

P=polyfit(t,M1',k);

yy=polyval(P,t);

p1=plot(t,yy,'-r','LineWidth',1.5,'MarkerSize',10)

M2=Ro(:,2);

P=polyfit(t,M2',k);

yy=polyval(P,t);

p2=plot(t,yy,'-b','LineWidth',1.5,'MarkerSize',10)

M3=Ro(:,3);

P=polyfit(t,M3',k);

yy=polyval(P,t);

p3=plot(t,yy,'-g','LineWidth',1.5,'MarkerSize',10)

M4=Ro(:,4);

P=polyfit(t,M4',k);

yy=polyval(P,t);

p4=plot(t,yy,'-y','LineWidth',1.5,'MarkerSize',10)

M5=Ro(:,5);

P=polyfit(t,M5',k);

yy=polyval(P,t);

p5=plot(t,yy,'-y','LineWidth',1.5,'MarkerSize',10)

M6=Ro(:,6);

P=polyfit(t,M6',k);

yy=polyval(P,t);

p6=plot(t,yy,'-y','LineWidth',1.5,'MarkerSize',10)

axis([1,15,-1,1])

set(gca,'Xtick',[1:15]);

set(gca,'XtickLabel',{'2';'4';'6';'8';'10';'12';'14';'16';'18';'20';'22';'24';'26';'28';'30'},'fontsize',22,'fontname','arial');

xlabel('days','fontsize',26,'fontname','arial')

ylabel('PRCC','fontsize',26,'fontname','arial')

hl=legend([p1,p2,p3,p4,p5,p6],'\fontsize{22}\it\lambda','\fontsize{22}\itp_{h}','\fontsize{22}\itd_{s}','\fontsize{20}\itd_{sh}','\fontsize{22}\itincubation period','\fontsize{22}\itp','Location','NorthWest')

set(hl,'Box','off','Orientation','horizon')

set(gca, 'Ytick', [-1:0.2:1])

grid on
